# MicroRNAs as Molecular Switches in Macrophage Activation

**DOI:** 10.3389/fimmu.2019.00799

**Published:** 2019-04-17

**Authors:** Graziella Curtale, Marcello Rubino, Massimo Locati

**Affiliations:** ^1^Department of Medical Biotechnologies and Translational Medicine, University of Milan, Milan, Italy; ^2^Humanitas Clinical and Research Center - IRCCS, Rozzano, Italy

**Keywords:** macrophages, microRNA, endotoxin, Toll-like receptor, TAM

## Abstract

The efficacy of macrophage- mediated inflammatory response relies on the coordinated expression of key factors, which expression is finely regulated at both transcriptional and post-transcriptional level. Several studies have provided compelling evidence that microRNAs play pivotal roles in modulating macrophage activation, polarization, tissue infiltration, and resolution of inflammation. In this review, we highlight the essential molecular mechanisms underlying the different phases of inflammation that are targeted by microRNAs to inhibit or accelerate restoration to tissue integrity and homeostasis. We further review the impact of microRNA-dependent regulation of tumor-associated macrophages and the relative implication for tumor biology.

## Introduction

Inflammation plays a critical role in host defense to invading microbial pathogens and is also essential for the successful repair of tissue damage (1). In this process, the involvement of multiple immune cells is critical. Macrophages are important component of the innate immune response, with a prominent role in host defense and clearance of foreign microorganisms, and in tissue healing (2). The inflammatory response is elicited by the recognition of pathogen-associated molecular patterns (PAMPs) or danger-associated molecular patterns by specific receptors expressed on macrophages (DAMPs), such as Toll like receptors (TLRs). These receptors elicit a signaling cascade that enhances phagocytic activity and activates the production of pro-inflammatory cytokines, chemokines, reactive oxygen and nitrogen species, and antimicrobial peptides (3). The efficacy of macrophage-mediated inflammatory responses relies on the coordinated expression of key proteins involved in macrophage activation and polarization processes, whose expression is finely regulated at both transcriptional and post-transcriptional levels (4–9). Evidence accumulated over the last decade suggests a prominent role for microRNAs (miRNAs) as key regulators of macrophage differentiation, infiltration, and activation. In addition to initial studies indicating the capability of miRNAs to modulate the magnitude of the innate immune response, participating as integral components of feedback loop regulatory mechanisms, which significantly shape the inflammatory response, recent studies have also established their role in tuning macrophage differentiation and polarization. In particular, a complex and highly regulated network of miRNAs exerting a pervasive regulation of inflammatory pathways by targeting multiple component of the TLR signaling pathway and thus affecting the profile of inflammatory cytokines induced downstream has been defined (10). Collectively taken, miRNAs can be viewed as a new regulatory layer of inflammatory reactions operating as intracellular effectors of well-known pro- and anti-inflammatory mediators, including PAMPs and DAMPs, inflammatory, and anti-inflammatory cytokines and macrophage polarizing factors (e.g., IFNγ, TGFβ, glucocorticoids, IL-4) (10–13). In this review, we summarize the most recent findings addressing the role of miRNAs in macrophage-mediated inflammatory response, with particular emphasis on the molecular pathways affected by miRNA-mediated regulation during macrophage polarization, bacterial infection, endotoxin tolerance, and tissue regeneration.

## miRNA Biogenesis and Mechanism of Action

Genome-wide sequencing approaches have lead to the discovery of non-coding RNAs (ncRNAs), which account for approximately 98% of the entire genome output, compared to the remaining 2% corresponding to protein-coding transcripts (14). Evolutionary studies have demonstrated that the increase in organisms complexity corresponds to a decrease in the abundance of protein-coding genes and a concomitant rise in the number of ncRNAs, indicating that regulatory RNA diversification has been critical to increase vertebrate complexity (15, 16). In this scenario, the most extensively studied class of ncRNAs is represented by miRNAs, short (20–24 nt in length) single-stranded RNA molecules which comprise 1–2% of all genes in worms, flies, and mammals (17, 18). According to information available in public repositories, at today 48885 mature different miRNAs have been reported in 271 species (miRBase 22, www.mirbase.org) (19). miRNAs essentially undergo the same regulatory mechanisms of any other protein-coding gene, being in normal conditions transactivated or silenced by specific transcription factors (4–7), affected by chromosomal deletions or amplifications (5, 8), and/or point mutations (9, 10). Moreover, their expression level is also affected by extensive epigenetic regulatory mechanisms, such as promoter methylation and histone modifications (20).

miRNAs are mainly transcribed by RNA polymerase II as primary miRNA (pri-miRNA) transcripts, with a local stem-loop structure and typically over 1 kb in length, in which the mature miRNA sequence is embedded (21). Following transcription, the pri-miRNA undergoes several steps of maturation. First, in the nucleus the RNAse III Drosha and its essential cofactor *DiGeorge syndrome critical region 8* (DGCR8) form a complex, called Microprocessor, that initiates the maturation process by cropping the pri-miRNA to release a small hairpin-structured miRNA precursor (pre-miRNA), of about 65 nucleotides in length (22). The pre-miRNA is then exported to the cytoplasm through an Exportin-5 (XPO5)-mediated system (22). There, a cytoplasmic RNAse III protein, called Dicer, generates the mature miRNA duplex, that is subsequently loaded onto an AGO protein (most commonly AGO2), to form the effector complex called RNA-induced silencing complex (RISC) (22, 23). miRNA-loaded RISC specifically recognizes short sequences of 6-7 nt, located in the 3' untranslated region (UTR) of target mRNAs, that are complementary to the so called “seed region” located in the 5' end of the miRNA molecule (18).

**Figure 1:**
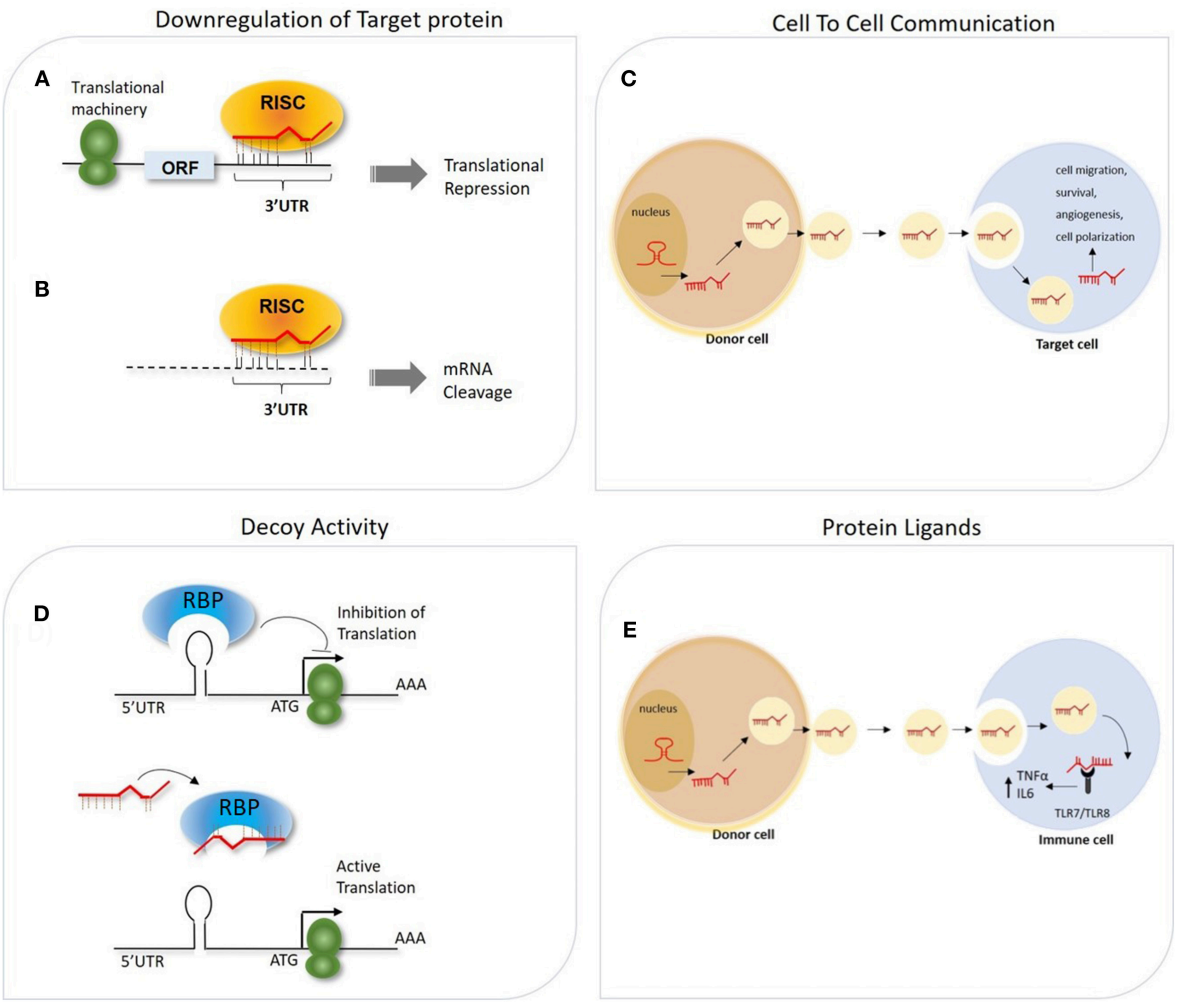
General overview of different mechanisms of miRNA-mediated gene regulation. **(A,B)** The association of miRNA-RISC complex to the 3′ UTR of mRNA target can lead to miRNA-dependent downregulation of the targeted gene through two potential mechanisms, depending on the degree of complementarity between the seed region and the 3′UTR: **(A)** Translation initiation inhibition **(B)** mRNA target degradation. **(C)** Secreted miRNAs within exosomes can act as intercellular messenger released from donor cells to regulate gene expression in the recipient cells. **(D)** miRNAs can interfere with the activity of RNA-binding proteins (RBPs) by pairing with the RBP itself and impeding the mRNA-RBP interaction. **(E)** miRNA can also be transported via exosomes from donor cells to recipient cells, in which these miRNAs function as TLR ligands.

miRNAs are implicated in most biological processes, being able to regulate post-transcriptionally the expression of hundreds of transcripts in the same cells. According to computational analysis based on evolutionarily conservation of miRNA-mRNA target pairing, more than 60% of human coding transcripts are predicted to be regulated by one or more miRNAs (24). The biological outcome of the miRNA-mRNA interaction is strongly affected by several factors, including the binding strength of such interaction (e.g., perfect or imperfect complementarity between the miRNA seed region and the relative target site), sequence features (e.g., site accessibility, RNA secondary structure) (25–28) and the relative abundance in the cell of distinct RISC cofactors (e.g., deadenylase complexes, dsRNA binding proteins), which is itself influenced by the cell identity and its activation conditions (29–31). It was primarily acknowledged that miRNAs act as repressors of gene expression by multiple mechanisms (Figure 1). These include their ability to impair mRNA stability by recruiting factors and enzymes involved in mRNA cleavage and degradation (i.e., endo/exo-nucleases, decapping enzymes, deadenylase), to interfere with protein translation by blocking the initiation or elongation steps, to sequester and segregate the target mRNA into processing bodies (P-bodies), and finally to function as RNA decoy by competing with RNA-binding proteins for their binding to a specific mRNA target (18, 32–34) (Figure 1). Independently by the specific mechanisms adopted, increasing evidence suggests that miRNAs may act as a buffer system against internal and external cell perturbations and confer robustness to biological processes by reinforcing transcriptional programs and attenuating the effects of aberrant transcription (35). To further add to the complexity of miRNA-mediated regulation, it has been showed that, in specific cell contexts (e.g., proliferation or cell cycle arrest) miRNAs are also capable of activating gene expression, directly or indirectly (36). The post-transcriptional regulatory functions of miRNAs is key to allow cells to rapidly respond to different cellular cues, thus representing an important component of cellular networks defining the cell state (35). Emerging evidence also indicates biological activities of miRNAs unrelated to their role of intracellular regulators of mRNA transduction. Exosome-derived miRNAs released by immune cells have been demonstrated to operate as extracellular soluble mediators with regulatory effects on adjacent and remote cells or tissues through endocrine or paracrine signaling (37, 38). Few studies also reported the ability of miRNAs to directly bind proteins in a sequence-specific manner. As examples, some miRNAs released by cancer cells have been shown to bind to TLR7 and TLR8, inducing a pro-metastatic inflammatory response (39), while miR-328 directly binds to the poly(rC)-binding protein hnRNP E2, which normally interacts with the 5′-UTR of CCAAT/enhancer binding protein alpha (C/EBPα) mRNA, causing the release of C/EBPα from hnRNP E2-mediated translational inhibition and the consequent increased expression of C/EBPα expression (40). These findings strongly point out the multi-faceted role of miRNAs in regulating important cellular processes. In the following sections, we will focus on the effect of such regulation on macrophage-mediated immune responses.

## Macrophages Polarization and miRNAs

**Figure 2:**
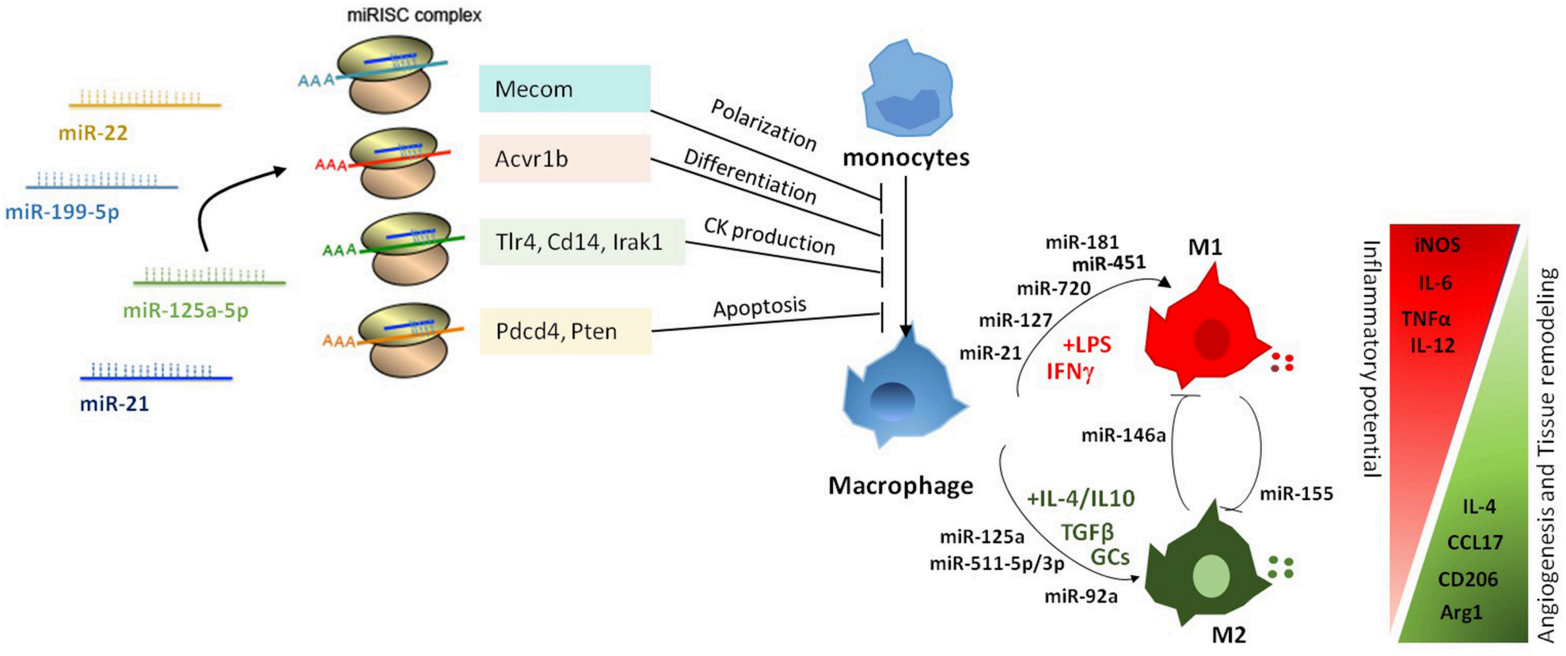
Pleiotropic role of miRNAs in the regulation of macrophage activation and polarization. miRNAs affect macrophage activation and differentiation by exerting a multiple regulation of sets of genes involved in different biological processes. Differential expression of miRNAs in macrophages also modulates macrophage polarization from a pro-inflammatory M1 to an anti-inflammatory M2 phenotype.

Macrophages possess a broad array of cell surface receptors, intracellular mediators, and essential secretory molecules for recognition, engulfment, and destruction of invading pathogens and also regulation of other type of immune cells and serve as sentinels of the immune system, sensing the presence of pathogens by means a variety of membrane anchored and cytosolic detectors. Macrophages participate in the inflammatory process by adapting their functional phenotype according to microenvironmental cues (41–43), and plasticity confers them the ability to coordinate host defense mechanisms to eliminate the pathogen and re-establish homeostasis in the host tissues (41, 44, 45). To efficiently induce protection from invading pathogens, macrophages mount an effective and balanced inflammatory response that mainly comprises four orderly stages: (a) recognition of foreign pathogens by pattern-recognition receptors (PRRs); (b) eradication of invading agents; c) resolution of inflammation through the involvement of suppressing cells and the release of anti-inflammatory mediators; e) tissue repair and restoration of tissue homeostasis. Macrophage remarkable heterogeneity results in the acquisition of an array of phenotypes and functional properties in response to different microenvironmental factors, and manifests as a spectrum of different functional states, oversimplified by the canonical dual distinction between classically- (M1) and alternatively-activated macrophages (M2) (5, 46–48). Classically activated macrophages respond to intracellular bacterial products (e.g., lipopolysaccharide, LPS) and several pro-inflammatory cytokines (including interferon gamma [IFNγ] and tumor necrosis alpha [TNF]) (8, 49). By contrast, the induction of alternative activated macrophages is favored by anti-inflammatory cytokines (IL-10, IL-4/IL-13), TGFβ, and glucocorticoids (GCs) (50) (Figure 2). M2-polarized macrophages are typically associated with immune response to parasites, wound healing and promotion of angiogenesis. They express tissue-remodeling and pro-angiogenic factors and inhibit the secretion of pro-inflammatory cytokines (51–53).

Genome-wide studies profiling transcriptional and epigenetic modifications reveal profound dynamic changes at gene loci associated with macrophage polarization, resulting in the coordinated action of distinct signaling pathways and transcription factors (8, 43, 54, 55). Several studies identified specific subsets of miRNAs differentially expressed under distinct polarizing conditions and investigated the impact of miRNA deregulation in macrophage polarization. A study by Zhang et al. (56) identified 109 miRNAs differentially expressed in human and murine M1- and M2-polarized macrophages, focusing in particular on miR-155, miR-181, and miR-451 in M1 macrophages, and miR-146a, miR-125a, and miR-145-5p in M2 macrophages (Figure 2). Subsequent studies confirmed the higher expression of miR-155 in M1-polarized macrophages and of miR-146a, miR-125b, and miR-127 in M2-polarizing conditions (9), and investigated their relationship with target genes involved in macrophage activation. In particular, miR-155 has been extensively studied in the context of macrophage polarization and inflammation. Its expression levels largely increased when macrophages were polarized to the M1 phenotype; whereas in M2-polarized macrophages miR-155 levels were strongly decreased (57). Interestingly, miR-155 was not just an M1 phenotypic marker, but actually had a role in driving macrophage polarization as its inhibition resulted in impaired M1 polarization and its overexpression induced a re-polarization toward an M1 phenotype of M2-macrophages (57). Later on, studies in miR-155 knockout (KO) mice demonstrated that miR-155 drives the inflammatory phenotype of M1-macrophages by regulating the expression of approximately 650 genes (58). Although the mechanism by which miR-155 directs macrophage M1 polarization has not been completely elucidated, evidence indicates it directly targets the IL-13 receptor α1 (IL13Rα1) thus interfering with STAT6 activation and indirectly regulates the expression of other M2-related genes, including CD23, DC-SIGN, CCL18, and SERPINE (59, 60). M1 polarization is also supported by miR-127 and miR-125b, which have been shown to target Bcl6 and IRF4, respectively, with consequent increased expression of pro-inflammatory cytokines (61, 62). In particular, inhibition of Bcl6 by miR-127 led to reduced expression of the phosphatase Dusp1 and increased phosphorylation of JNK, and its knockdown resulted in reduced expression of M1 signature genes and promoted the transcription of M2-related genes (61). Finally, overexpression of miR-720, a miRNA downregulated by M2 stimulation, decreased the expression levels of GATA3 a transcription factor important in M2 macrophage polarization, thus resulting in the inhibition of M2 polarization. Consistently, ectopic expression of GATA3 restored the M2 phenotype in miR-720 overexpressing macrophages and enforced expression of miR-720 inhibited pro-migration behavior and phagocytic ability of M2-polarized macrophages (63).

The first miRNA associated with M2 polarization was miR-146a. Enforced expression of miR-146a in peritoneal macrophages resulted in reduced levels of M1-marker genes (e.g., iNOs, CD86, TNF, IL-12 and IL-6), and increased production of M2-phenotype markers (e.g., Arg1, CCL17, CCL22 and CD206). In contrast, miR-146a knockdown promoted M1 macrophage polarization and diminished M2 macrophage polarization (64). Mechanistically, it was demonstrated that miR-146a modulated macrophage polarization at least in part by targeting Notch1, PPARγ, and inhibin βA subunit of activin A (INHBA) (65). Other miRNAs highly expressed in M2 macrophages are miR-511-3p, miR-223, and let-7c (9, 66), all of which have been shown to promote M2 polarization. miR-511-3p, which was found highly expressed also in tumor-associated macrophages (67), targets Rho-associated coiled-coil containing protein kinase 2 (Rock2), a serine-threonine kinase that phosphorylates IRF4 (67), thus supporting expression of M2-related genes. miR-223 targets the transcription factor Pknox1 and its overexpression led to the inhibition of LPS-dependent release of IL-1β and IL-6 (68), thus enhancing the alternative anti-inflammatory responses and limiting the pro-inflammatory activity of M1 macrophages (69). Similarly, enforced expression of let-7c reduced the expression of M1-related genes (i.e., iNOs and IL-12) and increased levels of M2 markers (*i.e*., FR-β), *via* targeting of P21 activated kinase 1 (PAK1) (70) and C/EBP-δ (71). Also relevant for macrophage polarization is the miR-23a/27a/24-2 cluster, which is downregulated by M1-type stimuli and upregulated by M2-type stimuli. Interestingly, enforced expression of either miR-23a or miR-27a promoted the expression of pro-inflammatory cytokines and the concomitant inhibition of M2-type cytokines by acting on different signaling pathways. MiR-23a activated the nuclear factor-kappa B (NF-κB) pathway by targeting TNF-inducing protein 3 (TNFAIP3), and by targeting JAK1 and STAT6 directly suppressed the activity of this signaling pathway and reduced the production of M2 type cytokines, while miR-27a showed the same phenotype by targeting interferon regulatory factor 4 (IRF4) and peroxisome proliferator-activated receptor gamma (PPARγ) (72). Altogether, the examples illustrated above show complex regulatory networks between miRNAs and transcription factors driving macrophage polarization, strongly candidating miRNAs as prominent regulatory elements in macrophage biology.

## miRNA as Molecular Determinants in Macrophage-Mediated Inflammatory Response

As discussed above, plasticity is a peculiar trait of macrophages that render them critical innate immune cells with versatile functions (73–75). Excessive and inadequate macrophage activation may lead to inefficient elimination of invading pathogens and contributes to self-tissue damage in inflammatory and autoimmune disorders (74, 76–78). Therefore, fine regulation of macrophage activation is needed during inflammatory and infectious conditions. Macrophage response to infectious agents is elicited by a pro-inflammatory environment, which further promotes macrophage microbicidal activity by inducing the transcriptional activity of genes belonging to the M1 program (79, 80). Therefore, M1 macrophage polarization is usually associated with protection during acute infectious diseases. It is now apparent that the regulation of miRNA expression in response to bacterial pathogens is a crucial part of the host mechanism against infections. miRNA-induced expression can increase or inhibit macrophages activity against infection (81). miRNAs have been recently recognized as important modulators of multiple signaling pathways activated or repressed along the different phases of the inflammatory response (82). Of note, it has also been reported that some bacterial pathogens reprogram macrophage polarization and induce specific M2 programs in macrophages, to evade the innate immune response (83, 84).

A number of inflammation-related miRNAs have been reported to be regulated in response to bacterial infections. Upregulation of miR-146a/b was observed in human monocytes infected by *Salmonella* (85), whereas the concomitant downregulation of let-7 family members (e.g., miR-98) was correlated with the upregulation of IL-10 (68, 86). Similarly, *Listeria monocytogenes* infection induced upregulation of miR-155, miR-146a, miR-125a-3p/5p, and miR-149 (87), and a miRNA expression profile performed in human macrophages infected with *M. avium* showed upregulation of miR-155, miR-146a/b, miR-886-5p, let-7, and miR-29a and concomitant decreased expression of miR-20a, miR-191, miR-378, miR-185 (88). More in details, let-7e and miR-29a downmodulated, respectively, caspases 3 and 7, thus regulating the apoptosis process after mycobacteria infection (88). miR-15a/16 have been reported as upregulated in bone marrow-derived macrophages in sepsis (89). Interestingly, it was demonstrated that miR-15a/16 support macrophage antibacterial activities as their deletion resulted in increased phagocytosis and mitochondrial reactive oxygen species production (89). A prominent role in controlling macrophage activation in inflammatory conditions is exerted by miR-155, which is widely expressed in immune cells and has a pleiotropic role in regulating both innate and adaptive immunity. In macrophages, miR-155 expression is induced by TLR agonists and pro-inflammatory cytokines through an AP-1 and NF-κB-mediated mechanism (81, 90, 91). Consistent with its pattern of expression, miR-155 acts as an early regulator of the inflammatory response, being able to inhibit the expression of negative regulators of the TLR signaling, including suppressor of cytokine signaling-1 (SOCS1) and Src homology-2 domain-containing inositol 5-phosphatase 1 (SHIP1) (92–94). miR-155 also indirectly enhances TNFα production by increasing TNFα mRNA half-life and translation (81). In primary macrophages infected by *Francisella* spp., miR-155 directly repressed the expression of SHIP1 and consequently enhanced the release of the pro-inflammatory response (95). As for other pro-inflammatory genes, miR-155 expression is then repressed by IL-10, a prominent anti-inflammatory cytokine induced at late time points by LPS stimulation, which operates as a negative regulator of the acute inflammatory phase. IL-10 inhibits miR-155 transcription in a STAT3-depedent manner and this inhibitory effect of IL-10 leads to an increase in the expression of SHIP1 (96).

Insights on the importance of miRNA-mediated regulation of macrophage response to bacterial infection were provided in particular by extensive studies performed on *Mycobacterium tubercolosis* (TB) infection. TB infection has dramatic effects on gene expression in host cells and this is associated with significant changes in a distinct panel of miRNAs (4 upregulated miRNAs: miR-24, miR-142, miR-155, miR212; 3 downregulated miRNAs: miR-19a, miR-202, miR-376a) (97, 98). Another study revealed that TB also induced the expression of miR-125b, which directly downregulated TNFα expression, thus resulting in the increase of TB pathogenicity (81). Similarly, upregulation of miR-32-5p expression observed in THP-1 monocytic cells after TB infection resulted in the reduction of inflammatory cytokine levels and a concomitant increase in the survival rate of intracellular mycobacteria. Conversely, the inhibition of miR-32-5p suppressed the intracellular growth of TB and promoted the expression of IL-1β, IL-6, and TNF (99). Furthermore, miR-23a-5p induction after TB infection also suppressed autophagy in infected macrophages through a mechanism that implicated the downregulation of TLR2 (100). Finally, TB downregulates let-7f with consequent upregulation of is target A20, a feedback inhibitor of NF-kB pathway (101). Indeed, let-7f overexpression increases the production of cytokines such us TNFα and IL-1β and diminishes TB survival (101).

## Regulatory Functions of miRNAs in the TLR Signaling Pathway

Macrophage activation requires recognition of various microbial components by means of specific families of pattern recognition receptors (PRRs), including Toll-like receptors (TLR) (102, 103). Several miRNAs have been shown to be upregulated in response to TLR ligands, and many of them directly target components of the TLR signaling system, revealing the involvement of miRNAs in feedback regulatory mechanisms. Over 10 years ago, a pioneering study documented the upregulated expression of miR-146a, miR-155, and miR-132 in the human monocytic cell line THP-1 upon treatment with pro-inflammatory stimuli, including the TLR agonists LPS and palmitoyl-3-cysteine-serine-lysine-4 (104), and later on with the identification as major miR-146a targets of TRAF-6 and IRAK1, key adaptor proteins of the TLR signaling cascade required for NF-κB activation, the first regulatory loop was revealed (105, 106). Since then, several TLR-responsive miRNAs have been identified, including miRNAs induced at early time points (e.g., miR-146a, miR-155, miR-9, miR-21, miR-147b, miR-181b) (90, 104, 107) as well as miRNAs regulated at later time-points by late-induced anti-inflammatory mediators (e.g., miR-146b, miR-187, the miR-125a~99b~let-7e cluster) (10–12). A subset of miRNAs downregulated by TLR stimuli have also been described (e.g., miR-92a, miR-29b, let-7i, miR-107, miR-27a^*^, miR-532-5p, and miR-322) (108–113).

An essential mediator of the inflammatory signaling pathway is the NF-κB family of transcription factors, which coordinates the expression of an array of genes involved in the inflammatory response (e.g., TNFα, IL-1β, IL-6, IL-12p40, cyclooxygenase-2). Notably, several of the miRNAs regulated by TLR agonists were directly controlled by the NF-κB-dependent pathway (e.g., miR-146a, miR-155, miR-9), and in most cases those miRNAs operated a feedback control of the NF-κB-dependent response by fine tuning the expression of key members of this pathway. For instance, miR-9 is early induced by TLR agonists *via* the MyD88-NF-κB-dependent pathway in human monocytes and neutrophils and directly targets the p50 precursor NF-κB1 (107). More recently, miR-322 was also reported as a negative regulator of NF-κB1 expression (110, 113). Finally, miR-147 expression is induced by TLR4 *via* NF-κB, which physically binds to miR-147 promoter region, and the induction of miR-147 in turn represses the release of pro-inflammatory cytokines (114).

## Pervasive Regulation of TLR Signaling Pathway by IL-10-dependent miRNAs

TLR triggering sets in motion a regulatory network of genes that tightly control the immune response by balancing pro- and anti-inflammatory signals. Negative regulation of TLR signaling by miRNAs represents an important step in setting this balance. This can be achieved by inhibiting the expression of genes required for LPS response, as discussed above, but also by inducing the expression of anti-inflammatory molecules, as in the case of miR-21, which indirectly increases IL-10 expression as a consequence of its targeted repression of PDCD4 (115).

**Figure 3:**
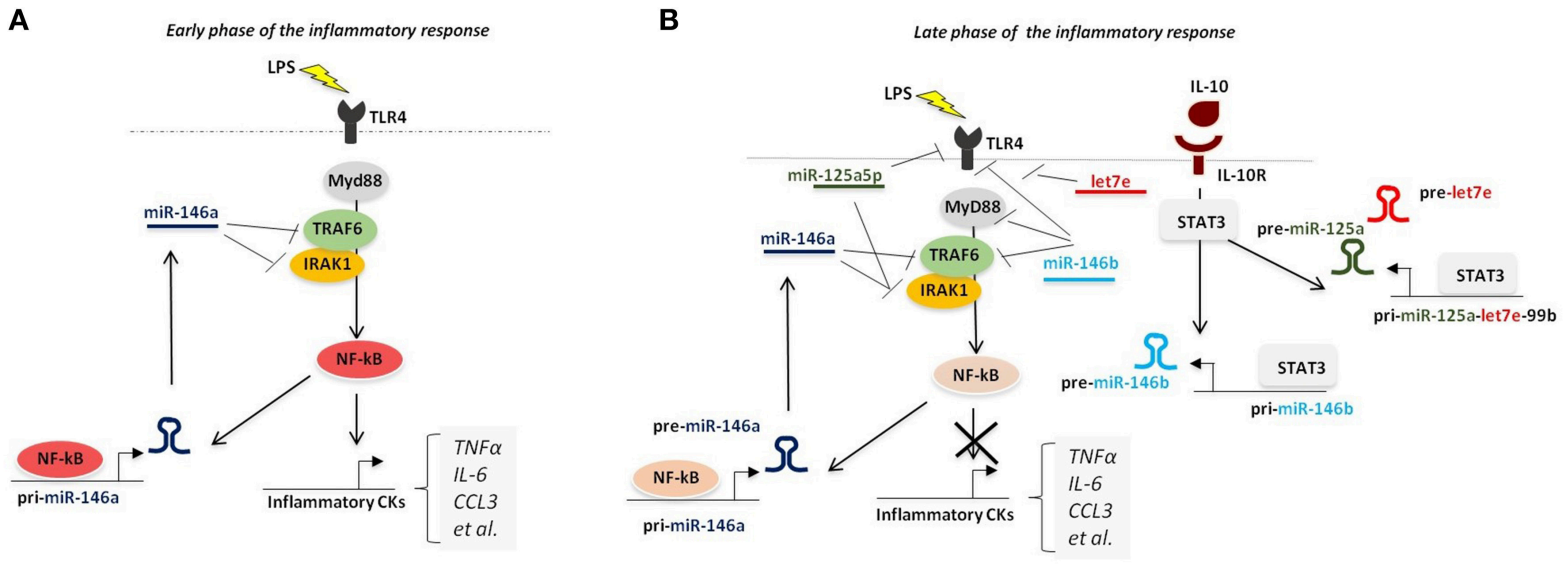
Pervasive regulation of TLR signaling pathway by miR-146a/b family and miR-125a~99b~let-7e cluster. **(A)** During overt inflammation binding of LPS to TLR4 induces the expression of NF-κB pathway, that ultimately leads to the release of pro-inflammatory cytokines (including TNFα, IL-6, CCL3, and IL-12). NF-κB also positively regulates the expression of miR-146a, which operates a negative feedback control of the NF-κB-dependent inflammatory response by repressing TRAF-6 and IRAK1. **(B)** During the late phase of the inflammatory response, production of IL-10 induces the expression of miR-146b and miR-125a~99b~let-7e cluster, *via* STAT3 binding to their promoters, thus resulting into downregulation of multiple components of TLR signaling pathway.

A small subset of miRNAs late expressed during the course of the inflammatory response was shown to be induced by IL-10 and, more strikingly, to mediate the IL-10-driven anti-inflammatory response. The first IL-10-dependent miRNA identified was miR-187, whose ectopic expression selectively reduced the production of TNFα, IL-6, and IL-12p40 by LPS-activated monocytes (11). This effect was at least in part mediated by direct targeting of NFKBZ, a master regulator of IL-6 transcription (11). Later on, miR-146b and the miR-125a~99b~let-7e cluster were also demonstrated to be late-induced after LPS challenge by an IL-10-dependent regulatory loop mediated by STAT3, the main transcription factor downstream IL-10. Whereas, miR-146a is early induced by LPS in both human and murine macrophages, miR-146b is selectively induced upon stimulation with the anti-inflammatory cytokine IL-10 *via* an NF-κB-independent pathway. Dependency of miR-146b expression on endogenous IL-10 was formally demonstrated in IL-10 KO mice, which were compromised in miR-146b but not miR-146b upon LPS triggering (12). miR-146b then exerts an anti-inflammatory activity by direct targeting the LPS receptor TLR4 and key adaptors/signaling molecules, including MyD88, TRAF6, and IRAK1 (12) (Figure 3). These findings also represent an example of two miRNA isoforms induced by different factors at different moments in the same cell type, suggesting that miR-146a/b might operate as a relay system to buffer the expression of pro-inflammatory genes induced by TLR4 triggering. The miR-125a~99b~let-7e cluster is also late induced by TLR agonists *via* the IL-10 dependent regulatory loop, and is counter-regulated by IFNγ (10), which promotes macrophage classic pro-inflammatory activation and chronic inflammation (116). miR-125a-5p and let-7e, but not miR-99b, enter the RISC complex in human primary monocytes and operate a pervasive negative regulation on the TLR signaling pathway by downregulating multiple components of the TLR signaling pathway, including receptors (e.g., TLR4, CD14) and signal transducers (e.g., IRAK1), with the resulting effect of a global suppression of downstream inflammatory cytokine production (10).

## Role of miRNA in Innate Immune Memory

Innate immune cells retain a memory of past stimulations and actions, allowing them to enhance (trained immunity) or repress (endotoxin tolerance; ET) the immune response when facing the same or different infectious agent (117, 118). At the base of both forms of innate memory is the establishment of a functional cell reprogramming resulting in the acquisition of new cellular properties, maintained long after the termination of the initial stimulus (119). The limited understanding of the molecular mechanisms behind this phenomenon points to an involvement of chromatin modifications and inducible regulatory molecules, such as miRNAs, which shape the different transcriptional programs and outcomes that characterize ET (120) and trained immunity (121).

During ET, monocytes and macrophages display reduced response to subsequent challenges after they have been exposed to low concentrations of endotoxin (111, 112). ET is a dynamic process that relies on the action of several negative regulatory loops resulting in repression of pro-inflammatory cytokines and chemokines (e.g., TNFα, IL-6, IL-12, CCL3, CXCL8, CCL2), that are transcriptionally silenced (that is, tolerized) upon LPS-re-exposure and concomitant upregulation of anti-inflammatory cytokines (e.g., IL-10, TGFβ, scavenging receptors (e.g., MARCO; CD64 and CLEC4a) and a variety of anti-microbial genes (e.g., RNASET2, FPR1) (112). We highlighted the impact of miRNAs in regulating TLR signaling pathways. Not far from the demonstration of miRNA-mediated regulation of the inflammatory response, further evidence suggested that specific miRNAs indeed play a role in the development of LPS tolerance (122). miR-146a was the first miRNA described as upregulated in tolerant THP-1 monocytic cells able to partially induce LPS desensitization (123). This effect has been related to its ability to downregulate the NF-κB pathway by acting on TRAF6 and IRAK1, and is consistent with evidence demonstrating high levels of miR-146a and impaired NF-κB activity in endotoxin-tolerant murine macrophages and human monocytes (124–126). Similar evidence has also been reported for miR-181b, which contributed to the downregulation of IL-6 after LPS exposure (127), and miR-155 (81, 90, 127). miR-155 expression is regulated by the phosphoinositide-3 kinase (PI-3K)-AKT kinase signaling pathway, which has a well-documented role in controlling macrophage sensitivity and ET (94, 128–130). Androulidaki et al. (94) demonstrated that the state of LPS tolerance was at least in part dependent on the regulation of let-7e and miR-155 expression by AKT1, which induced the former and suppressed the latter (94). As discussed, these miRNAs have opposite roles in controlling the inflammatory reaction, with let-7e inhibiting the inflammatory response by direct targeting of TLR4 (10, 94) and miR-155 promoting macrophage sensitivity to LPS response by SOCS1 downregulation (94). Of note, miR-155 is one of the few miRNAs induced not only by LPS, but also by the TLR3 agonist double stranded RNA and by IFNβ through TNF autocrine signaling (81, 90), and miR-155 knock-in mice are highly susceptible to LPS shock due to high levels of TNF (81, 90, 131).

Some miRNAs are differentially expressed during ET, but are not expressed or are late induced in LPS-primed macrophages. Among them miR-146b, (132), miR-125a~99b~let-7e cluster (10), miR-222 (133) and miR-511-5p (13). A prominent role in ET elicited by different anti-inflammatory stimuli (e.g., IL-10, GCs, TGFβ) was demonstrated for miR-146b (132), miR-125a~99b~let-7e cluster (10), and miR-511-5p (13). MiR-146b is late induced by LPS and higher expressed in monocytes tolerized by IL-10 and TGFβ. Regulation of miR-146b expression modulated ET in monocytes (132). Similarly, miR-125a~99b~let-7e cluster was found expressed at high levels in LPS tolerant monocytes. Of note, ET rescue by IFNγ, a cytokine known to suppress expression of miR-125a~99b~let-7e cluster, was impaired in cells overexpressing miR-125a~99b~let-7e cluster (10), indicating that IFNγ ability to prevent induction of LPS tolerance is at least in part mediated by the inhibition of this miRNA cluster (10). Similarly, miR-222 expression increased late during LPS response and is counteracted by IFNγ. In a recent paper published in 2018 (133). Inhibition of miR-222 was also shown to reduce the duration and magnitude of ET, by restoring the expression of tolerized genes, such as IL-6 and IL-12p40 at levels comparable to those observed in non tolerized cells. Differently from other miRNAs involved in ET regulation, miR-222 did not modulate expression of ET genes by regulating TLR4 signaling. Instead, it targeted BRG1, a catalytic subunit of the chromatin remodeling complex SWI/SNF, recruited to the promoters of late LPS-response genes to induce their transcription. Thus, miR-222 represents a paradigmatic example of the existence of a crosstalk between miRNAs and chromatin modifications, both important components of the mechanistic framework that is at the base of short-term memory in ET.

Whereas, the role of miRNA in ET is well established, less defined is their relative contribution in the other form of innate immune memory, that is trained immunity. A distinguishing feature of trained innate immune cell is its ability to mount a qualitatively different- and to some extent quantitatively stronger- transcriptional response compared to untrained cells when rechallenged with infectious or danger signal (134). miRNAs known as activators of the inflammatory response, such as miR-155, might also contribute to trained immunity, possibly through the repression of phosphatases or other negative regulators. Further studies are needed to define which miRNAs induced during trained immunity play an active role in initiating and sustaining the hyper- sensitive state of trained cells.

## Role of miRNA in Macrophage Anti-Inflammatory and Tissue Healing Activities

The functional and phenotypic plasticity of macrophages become particularly apparent during the resolution of inflammation, which is initiated with the clearance of apoptotic neutrophils (efferocytosis) by tissue resident macrophages and their switch from a pro- to an anti-inflammatory phenotype (135–137). In this biological setting, miRNAs have been described as part of negative regulatory loops that keep inflammation in check by promoting production of anti-inflammatory mediators, tissue healing, and return to homeostasis (138, 139). In an *in vivo* murine model of peritonitis, treatment with resolving D1 (RvD1), an endogenous lipid mediator generated during the resolution phase of acute inflammation, regulated resolution indices and controlled specific miRNA expression in exudates. (140). In particular, RvD1 upregulated miR-21, miR-146b, and miR-219, and downregulated miR-208a (140), and miR-21 was candidate as a novel component of a RvD1-miRNA circuit (140). Two other studies further confirmed the key role for miR-21 in controlling inflammation and promoting the switch from a pro-inflammatory to a pro-resolving phenotype of macrophages in relation to different anti-inflammatory stimuli. It was demonstrated that miR-21 downregulates the translation of the pro-inflammatory tumor suppressor programmed cell death 4 (PDCD4), an inhibitor of IL-10 (141), and this miR-21/PDCD4/IL-10 circuit was shown to play an important role in efferocytosis (142). Interestingly, in addition to PDCD4, miR-21 also promoted downregulation of PTEN and GSK3, with consequent inhibition of NF-kB and AP-1 activity and TNF production, thus bolstering the anti-inflammatory response (139, 141–143). In a model of sepsis, increase circulating levels of miR-466l were detected (144). This miRNA is early expressed in polymorphonuclear neutrophils, where it promotes inflammation, and then is induced at later time points in macrophages engaged by apoptotic neutrophils, and in macrophages it contributes to resolution by promoting efferocytosis (144). Interestingly, the presence of miR-466l was also confirmed in septic patients. Further studies are required to fully disclose the role of miRNAs in the resolution of inflammation and to evaluate their potential for the development of novel therapeutic approaches for inflammatory diseases.

## Role of miRNA in Tumor- Associated Macrophage Biology

Tumor infiltrating myeloid cells are educated by the tumor milieu and exert a number of pro-tumoral functions, ranging from promotion of tumor growth, to angiogenesis and immunosuppression (145–147). The two major myeloid cells associated with cancer-related inflammation are tumor-associated macrophages (TAM) and myeloid-derived suppressor cells (145). A detailed summary of miRNA-mediated regulation of myeloid-derived suppressor cells in the context of cancer-related inflammation has been recently reviewed elsewhere (20, 147). Here we focus on the participation of miRNAs in regulatory networks controlling the function of TAM, which are a double edge sword as they usually exert pro-tumoral functions but are potentially endowed with anti-tumoral activities. This crucial balance tightly depends on the macrophage activation status. As miRNAs modulate macrophage polarization in inflammatory conditions, it is not surprising that they also have been also implicated as intracellular determinants of the biology of tumor-educated macrophages (148, 149).

It has been reported that miRNAs are differentially expressed in myeloid cells during metastatic tumor progression in mouse models of melanoma and breast cancer, two biological contexts where the link between TAM and tumor progression and the relative molecular mechanisms are well-established. Colony-Stimulating Factor-1 (CSF-1) is a growth factor which modulates macrophage survival and functions during inflammation through the regulation of the transcription factor ETS2 (150–152). Genetic deletion of CSF-1 resulted in reduction of mammary TAMs and in lower incidence of lung metastasis in *in vivo* mammary tumor mouse models (153), deletion of ETS2 in macrophages resulted in reduced metastatic tumor burden (153) and its elevated expression has been correlated with human breast cancer (154). In TAM the CFS-1/ETS-2 pathway was associated with an oncogenic miRNA expression signature, including miR-223, miR-21, miR-29a, and miR-142-3p. These oncogenic miRNAs likely promote macrophage pro-tumoral functions, including tumor cell proliferation and angiogenesis, as miR-21 and miR-29a target genes involved in M1-polarization and anti-angiogenic regulators (155). Further evidence suggesting that endogenous miRNAs may exert important roles in controlling the polarization and function of TAM was obtained in a transplanted breast cancer mouse model, where miR-146a and miR-222 were significantly downregulated, and this was associated with the upregulation of the NF-κB p50 subunit (156). Interestingly, inhibition of miR-146a resulted in decreased expression of M2 macrophage genes in TAM and reduced tumor growth *in vivo*, while overexpression of miR-222 reduced macrophage recruitment by targeting CXCL12 and CXCR4 (156). Similarly, in agreement with the aforementioned role of mir-155 in negative regulation of M2 polarization, miR-155 knockdown in myeloid cells accelerated spontaneous breast cancer development by impairing macrophage classical activation, with consequent imbalance toward a pro-tumoral microenvironment which favored the skew of tumor-associated immune cell toward an M2/Th2 response (157). miR-511-3p is another M2-associated miRNA, that was found to be also highly active in TAM, where it triggers a negative feedback response that inhibits tumor growth and attenuates TAM pro-tumoral genetic programs (67). Finally, exosome-secreted miRNAs have been described as an alternative mechanism adopted by TAM to modulate breast cancer invasion and metastasis, as miR-223 was detected within macrophage exosomes and was shown to promote invasiveness of breast cancer cells *via* the targeting of MEF2C-β-CATENIN pathway (158).

## Conclusions

In the last decade, several efforts have been made to shed light into the molecular mechanisms driving the inflammatory response, in all its facets. miRNAs have been demonstrated to be pivotal players actively participating in the modulation of the early phase as well as the resolution of inflammation. Recent findings on their involvement in chronic inflammatory conditions, sepsis, and tumors strongly encourage the development of new miRNA-based therapeutic strategies.

## Author Contributions

All authors listed have made a substantial, direct and intellectual contribution to the work, and approved it for publication.

### Conflict of Interest Statement

The authors declare that the research was conducted in the absence of any commercial or financial relationships that could be construed as a potential conflict of interest.
